# Sports activity and the use of cigarettes and snus among young males in Finland in 1999-2010

**DOI:** 10.1186/1471-2458-12-230

**Published:** 2012-03-22

**Authors:** Ville M Mattila, Susanna Raisamo, Harri Pihlajamäki, Matti Mäntysaari, Arja Rimpelä

**Affiliations:** 1 Research Department, Centre of Military Medicine, 00101 Helsinki, Finland; 2 Department of Orthopedics and Traumatology, Tampere University Hospital, 33100 Tampere, Finland; 3 School of Health Sciences, University of Tampere, 33100 Tampere, Finland

**Keywords:** Young people/youth, Tobacco use, Snus, Smoking, Sports, Physical activity

## Abstract

**Background:**

Studies of the relationship between sports activity and smoking among adolescents and young adults report contradictory results. We examined the association between sports activity (intensity and type of sport) and the current use of snus (Swedish snuff), cigarette smoking, and the combined use of cigarettes and snus (dual use) among young males in Finland.

**Methods:**

Data were collected from 16,746 male conscripts who completed a survey during the first days of their conscription during the years 1999-2010 (median age 19 years, response rate 95%). Main outcome measures were self-reported daily/occasional use of snus, cigarette smoking, and dual use. The association between sports activity, type of sport, and several sociodemographic background variables was assessed using logistic regression analysis.

**Results:**

Over the study period (1999-2010), the prevalence of cigarette smoking decreased from 42% to 34%, while snus use increased from 5% to 12%, and dual use increased from 7% to 13% (*p *< 0.001). Compared with no physical activity, regular competitive sports activity (defined as high-intensity sports activity) was positively associated with use of snus (odds ratio [OR] 10.2; 95% confidence interval [CI]: 7.8-13.5) and negatively with cigarette smoking (OR 0.2; 95% CI: 0.1-0.3). When stratified by type of sport in multivariate models, ice hockey was most strongly associated with snus use (OR 1.6; 95% CI: 1.4-1.9) and dual use (OR 2.0; 95% CI 1.8-2.3) compared with those not playing ice-hockey, followed by other team sports for snus use (OR 1.5; 95% CI: 1.3-1.8) and dual use (OR 1.8; 95% CI: 1.6-2.0) compared with those not participating in other team-sports.

**Conclusions:**

Our results show a clear association between snus use and intensity and type of training. Team sports were associated with increased use of snus and dual use compared with no participation in team sports. These findings should be acknowledged when planning and implementing preventive strategies.

## Background

Studies on the relationship between physical activity and smoking among adolescents and young adults report contradictory results [1]. Some findings indicate that participation in sports and physical activity is a protective factor against smoking initiation. For example, in a longitudinal cohort study of the United States of America (USA) high school students, higher levels of physical activity reduced the risk of smoking during adolescence [2]. In a survey among university students in Italy, Bergamaschi and colleagues found that youth who engaged in physical activity smoked less than their inactive peers [3]. Moreover, smokers who took part in sports smoked fewer cigarettes per day [3].

On the other hand, other studies have reported opposite or more inconclusive results. Using the data from the 2003 Health Survey for England, Poortinga revealed that in an English adult population aged 16 years and over, those who were physically active were more likely to smoke and to drink heavily [4]. A Danish study from 2008 among 16- to 20-year-olds reported an inverse association between physical activity and smoking, but the association disappeared when study covariates were taken into account [5]. A recent cross-sectional study based on the Stockholm Public Health Survey demonstrated that sedentary lifestyle is strongly associated with smoking and the combined use of snus (Swedish snuff) and cigarettes among 18- to 84-year-old Swedish men and women [6].

The relevance of certain types of sport to the differences in youth tobacco use has also been highlighted; a previous Finnish study examining the trends between physical activity and snus use among 16- to 18-year-old boys suggested that snus use is common in team sports, especially ice hockey and floor ball [7]. In the USA, smokeless tobacco use has long been linked to baseball [8]. Several explanations have been suggested, including the less damaging effect of snus on oxygen intake compared to cigarette smoking, other health-related factors, beliefs, and socio-demographic factors [1]. While the positive associations of physical activity with alcohol consumption are explained by participation in organized sport (sport hypothesis), there has been no evidence to support the sport hypothesis with regard to smoking [4].

In Finland, approximately about one-fourth of the population aged 15 to 64 years (22% of men, 16% of women) are daily smokers [9]. The prevalence of daily smoking in adolescents aged 12 to 16 years has declined over the last decade, but the rates among 18-year-old males have remained fairly stable [10]. The sale of snus was prohibited in the entire European Union (EU) region in 1992, and when joining the EU in 1995, Finland applied for a total ban on snus sales. Sweden, a neighbouring country of Finland, was the only country in the EU granted special exemption to manufacture snus. Despite the sales ban in Finland, the popularity of snus use, particularly among 16- and 18-year-old boys, has increased [11]. Based on the latest nationwide survey of adolescent health and health behaviours in Finland, 14% of 18-year-old boys and 12% of 16-year-old boys were current snus users in 2011 [12]. Similar increasing trends in snus use have been reported in the USA [13]. Of Finnish men aged 15 to 24 years, approximately 6% have used snus [9].

Although several studies have investigated the relationship between physical activity and cigarette smoking, evidence for an association between snus use or the combined use of cigarette smoking and snus (dual use) and sports activity is scarce or largely inconclusive. Moreover, a gap exists in the literature regarding the association between different types of sport and the intensity levels of physical activity, and differences in tobacco product use [14]. Previous studies suggest that people with high sports intensity prefer snus to smoking [7,15], with smaller effects on respiratory function being a plausible explanation [7,15]. Such knowledge would be valuable for those working toward smoking prevention and cessation, allowing them to tailor programs more efficiently.

The aim of this study was to examine the association between sports activity and snus use, cigarette smoking, and dual use of these tobacco products among young males in military service. We evaluated the changes in these relationships over eight consecutive surveys conducted during 1999-2010.

## Methods

The study sample comprised healthy young men (median age 19 years, range: 18-29 years) entering their obligatory military service in Finland, which is completed by 80% of the age group. We used data obtained from the nationwide Finnish Conscript Health Survey, which is aimed at exploring conscript health and health-related lifestyles. The conscripts were asked to answer the survey questionnaire on the first days of their military service. The Medical Ethics Committee of the Finnish Centre of Military Medicine approved the study. Of the 28 Finnish garrisons, 10 were randomly selected and all respondents from 6 years were included: 1999 (n = 843), 2001 (*n *= 1473), 2002 (*n *= 1870), 2003 (*n *= 1678), 2005 (*n *= 1788), 2006 (*n *= 1707), 2007 (*n *= 1883), 2008 (n = 1752), 2009 (n = 1842), and 2010 (n = 1944). The survey was not conducted in 2000 and 2004. The total number of respondents was 16,746 (response rate 95%). The questionnaires have always been anonymous, and contain no personal identifiers.

The main outcome variable of interest in our study was self-reported daily/occasional use of snus, cigarette smoking, or dual use (combined cigarette smoking and snus use). The question was posed as follows:"Do you smoke cigarettes/use snus daily or occasionally?". The main outcome variable was categorized into four mutually exclusive groups regarding tobacco use: no use of tobacco or snus, exclusive use of snus, exclusive cigarette smoking, and dual use. Daily and occasional users were combined because of the small number of daily snus users.

### Background variables

A total of three variables obtained from the questionnaire were used to describe the conscripts' sociodemographic background. Urbanization level of residence was determined by the population density under five categories: capital area (Helsinki and adjoining cities), city/large town (population over 100,000), small town, village (densely populated area in rural municipalities), and sparsely populated rural municipality (isolated homestead in rural municipalities). Childhood family composition was categorized into nuclear (two biological parents) and nonnuclear (other than two biological parents). Three categories of level of education achieved were used: low (comprehensive school), middle (vocational school), and high (upper secondary school or university).

Respondents' sports behaviours were described by the following 12 variables (see Table 1): Conscripts were asked how they perceived their physical fitness (poor, average, good, or excellent) and about the intensity of their leisure-time physical activity during the half year period prior to their military service (no physical activity, light activity < 5 h/wk, moderate activity 5 h/wk, regular sports activity [high activity]). Participation in the most common sports activities in Finland (walking, jogging/running, swimming, weight training, team sports, skiing, ice hockey, and downhill skiing) performed at least once a week was also assessed. The most common team sports in Finland are football, floorball, volleyball, and basketball.

**Table 1:** Characteristics of the respondents and separate bivariate age-adjusted logistic regression models for snus use, cigarette smoking, and dual use

Characteristics	n (%)	OR for snus use	OR for cigarette smoking	OR for dual use
*Intensity of sport activity*				
Leisure-time sport activity				
no physical activity	4267 (27)	1	1	1
light activity < 5 h/wk	5837 (36)	1.7 (1.2-2.2)	0.7 (0.6-0.7)	0.8 (0.7-0.9)
moderate activity 5 h/wk	4320 (27)	3.6 (2.8-4.8)	0.3 (0.3-0.4)	0.8 (0.7-1.0)
regular competitive sports training	1638 (10)	10.2 (7.8-13.5)	0.2 (0.1-0.3)	1.3 (1.1-1.6)
Self-reported physical fitness				
Poor	2500 (15)	1	1	1
Average	6712 (41)	1.0 (0.7-1.4)	1.0 (0.9-1.1)	0.9 (0.8-1.1)
Good	5774 (36)	2.5 (1.9-3.3)	0.5 (0.4-0.6)	1.0 (0.9-1.2)
Excellent	1289 (8)	6.1 (4.5-8.3)	0.2 (0.1-0.3)	0.7 (0.6-0.9)
**Sport type**				
Weekly walking				
No	11402 (70)	1	1	1
Yes	4865 (30)	0.5 (0.4-0.6)	1.1 (1.0-1.2)	0.7 (0.6-0.7)
Weekly running				
No	11806 (73)	1	1	1
Yes	4460 (27)	1.5 (1.2-1.7)	0.4 (0.4-0.5)	0.6 (0.5-0.6)
Weekly swimming				
No	15282 (94)	1	1	1
Yes	984 (6)	0.8 (0.5-1.1)	0.8 (0.7-0.9)	1.0 (0.8-1.2)
Weekly cycling				
No	14063 (87)	1	1	1
Yes	2203 (14)	0.8 (0.6-1.0)	0.6 (0.6-0.7)	0.5 (0.4-0.6)
Weekly team sports (not ice hockey)				
No	12031 (74)	1	1	1
Yes	4237 (26)	2.5 (2.1-2.9)	0.7 (0.6-0.7)	1.7 (1.5-1.9)
Weekly weight training				
No	12458 (77)	1	1	1
Yes	3809 (23)	2.0 (1.7-2.3)	0.6 (0.6-0.7)	1.2 (1.1-1.3)
Weekly combat training				
No	15307 (94)	1	1	1
Yes	959 (6)	1.2 (0.9-1.6)	0.7 (0.6-0.8)	1.1 (0.9-1.3)
Weekly skiing				
No	14560 (92)	1	1	1
Yes	1328 (8)	0.9 (0.7-1.2)	0.5 (0.4-0.6)	0.4 (0.3-0.5)
Weekly ice hockey				
No	12662 (80)	1	1	1
Yes	3227 (20)	2.1 (1.8-2.3)	0.8 (0.8-0.9)	2.0 (1.8-2.2)
Weekly downhill skiing				
No	14112 (89)	1	1	1
Yes	1775 (11)	1.0 (0.8-1.4)	1.2 (1.1-1.4)	1.4 (1.2-1.6)
***Sociodemographic background***				
Urbanization level of residence				
Capital area (Helsinki and adjoining cities)	3447 (21)	2.6 (1.8-3.6)	0.8 (0.7-0.9)	1.4 (1.1-1.7)
City/large town	6811 (41)	2.2 (1.6-3.1)	0.9 (0.8-1.0)	1.7 (1.5-2.1)
Small town	2541 (15)	1.5 (1.1-2.3)	1.1 (0.9-1.2)	1.3 (1.1-2.1)
Village	2080 (12)	1.6 (1.1-2.4)	0.9 (0.8-1.1)	1.3 (1.0-1.7)
Sparsely populated rural municipality	1776 (11)	1	1	1
Childhood family composition				
Nuclear	14041 (85)	1	1	1
Nonnuclear	2570 (15)	0.8 (0.6-1.0)	1.4 (1.3-1.6)	1.3 (1.1-1.3)
Level of education				
High	2326 (14)	1.4 (1.1-1.8)	0.3 (0.2-0.3)	0.5 (0.4-0.5)
Middle	6522 (39)	0.9 (0.7-1.2)	0.7 (0.6-0.8)	0.6 (0.5-0.7)
Low	7848 (47)	1	1	1

Due to missing values, the sum of the respondents in different categories is less than the total number of 16,746 Reference category marked with 1

### Statistical analysis

At the first stage, age-adjusted logistic regression models were conducted for each tobacco variable (snus use, smoking, and dual use) and all independent variables. In all cases, non-use was a reference category. Odds ratios (OR) were estimated with 95% confidence intervals (95% CI).

Next, fully-adjusted models were conducted. Due to the fact that sociodemographic status, and sports type and intensity are associated, we entered sociodemographic background variables, the intensity of leisure-time physical activity, and self-perceived physical fitness into the age-adjusted models. For all models Nagelkerke R^2 ^varied from 0.034 - 0.139 (Table 2).

**Table 2:** Nagelkerke R square for multivariate models presented in Table 3

	Nagelkerke R square for snus use models	Nagelkerke R square for cicarette smoking models	Nagelkerke R square for dual use models
**Model**			
Weekly walking	0.104	0.133	0.039
No			
Yes			
Weekly running	0.040	0.139	0.099
No			
Yes			
Weekly swimming	0.101	0.132	0.101
No			
Yes			
Weekly cycling	0.034	0.041	0.135
No			
Yes			
Weekly team sports (not ice hockey)	0.101	0.132	0.046
No			
Yes			
Weekly weight training	0.036	0.132	0.100
No			
Yes			
Weekly combat training	0.132	0.034	0.040
No			
Yes			
Weekly skiing	0.135	0.101	0.105
No			
Yes			
Weekly ice hockey	0.134	0.050	0.036
No			
Yes			
Weekly downhill skiing			
No			
Yes	0.137	0.099	0.100

Differences in two-way tables in tobacco use prevalences at different points in time were assessed using a chi-square test with the level of significance defined as *p *= 0.05.

We also explored whether the associations between tobacco product use and sports activity changed over time. To test for associations over time, the data were divided into three datasets based on survey year (1999-2003, 2005-2007, and 2008-2010), and separate logistic regression models were calculated for time period for each tobacco variable.

Logistic regression analyses were performed only for respondents who provided answers to every question and thus respondents with incomplete answers were excluded from the analysis. The frequency of missing values for the independent variables varied from 1% to 3% (Table 1), while the frequency of missing values for the dependent variable was 4.9%.

## Results

**Figure 1:**
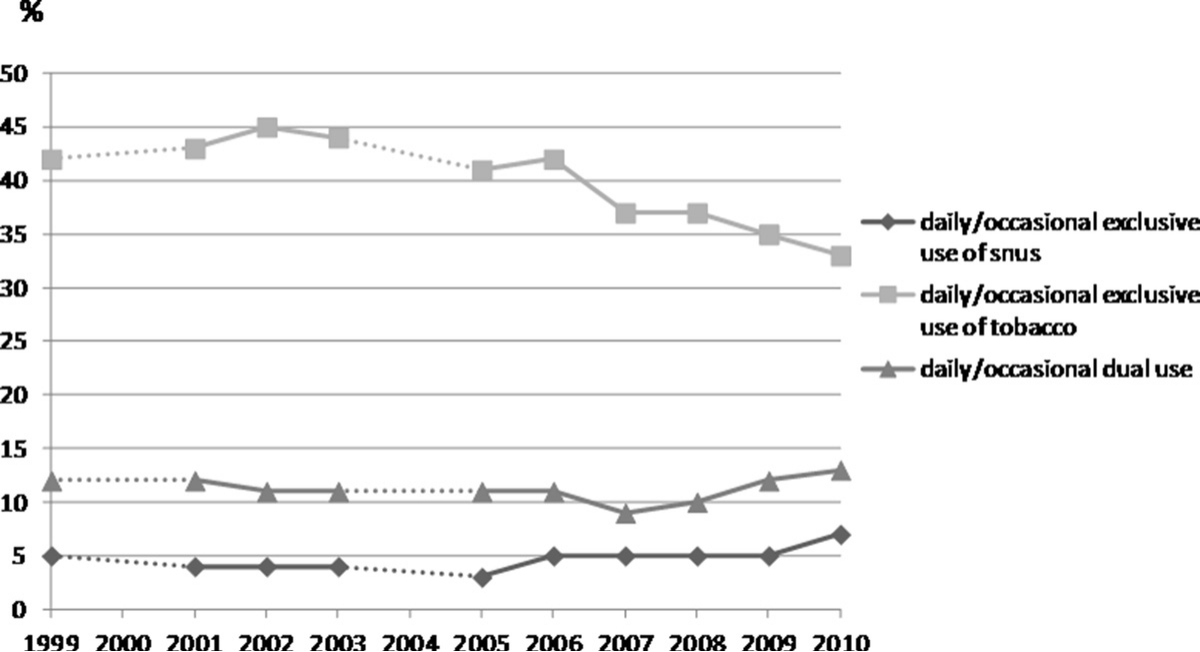
**The frequency of smoking, snus use and dual use among young males in Finland in 1999-2010**.

Of the 16,746 respondents, 735 (4.4%) reported exclusive daily/occasional snus use, 6178 (36.8%) exclusive daily/occasional cigarette smoking, and 1771 (10.5%) daily/occasional dual use. Figure 1 shows the trend in tobacco product use from 1999 to 2010. Cigarette smoking decreased from 41.8% in 1999 to 34.2% in 2010, while both snus use and dual use increased from 4.9% and 12.1% (1999) to 6.8% and 13.4% (2010), respectively (*p *< 0.001).

In the age-adjusted logistic regression analysis, sociodemographic background was significantly associated with the use of tobacco products (Table 1). The odds ratios for the use of snus and dual use were significantly higher among those living in the capital city area and in large cities than in rural areas (OR 2.6; 95% CI: 1.8-3.6). In addition, the odds ratio for smoking decreased with higher educational status (OR 0.3; 95% CI 0.2-0.3 in high education group compared with low education group). Those reporting regular competitive sports activity had an increased odds ratio for use of snus (OR 10.2; 95% CI: 7.8-13.5) and a decreased odds ratio for cigarette smoking (OR 0.2; 95% CI: 0.1-0.3) compared to those reporting no sports activity at all. A corresponding association was observed in self-reported physical fitness (Table 1). Team sports, ice hockey, and weight training were positively associated the most with snus use. Downhill skiing was the only sports activity positively associated with cigarette smoking while all other types of sport except walking were negatively associated with cigarette smoking. (Table 1)

When sociodemographic background, intensity of sports activity, and self-reported physical fitness were controlled for in the multivariate logistic regression model (Table 3), ice hockey was positively associated with snus use (OR 1.6; 95% CI: 1.4-1.9) and dual use (OR 2.0; 95% CI 1.8-2.3) compared to those not engaged in ice hockey. Other team sports participation was also positively associated with snus use (OR 1.5; 95% CI: 1.3-1.8) and dual use (OR 1.8; 95% CI: 1.6-2.0) compared to those not participating in team sports. Ice hockey and other team sports were not associated with cigarette smoking (OR 1.0; 95% CI: 0.9-1.1 for ice hockey and OR 1.0; 95% CI: 0.9-1.1 for team sports). Cycling and skiing were negatively associated with snus use, cigarette smoking, and dual use compared to those not engaged in cycling or skiing. Downhill skiing was the only sport positively associated with cigarette smoking in multivariate models (OR 1.4; 95% CI: 1.2-1.6; Table 3).

**Table 3:** Adjusted* odds ratios (ORs) and 95% confidence intervals (CIs) for snus use, cigarette smoking, and dual use of snus and cigarettes by type of weekly sport type

	OR for snus use (95% CI)	OR for cigarette smoking (95% CI)	OR for dual use (95% CI)
**Sport type**			
Weekly walking			
No	1	1	1
Yes	0.6 (0.5-0.8)	1.1 (1.0-1.2)	0.7 (0.6-0.8)
Weekly running			
No	1	1	1
Yes	0.9 (0.7-1.1)	0.7 (0.6-0.7)	0.6 (0.6-0.7)
Weekly swimming			
No	1	1	1
Yes	0.7 (0.4-0.8)	1.0 (0.8-1.1)	1.0 (0.8-1.3)
Weekly cycling			
No	1	1	1
Yes	0.7 (0.5-0.9)	0.7 (0.7-0.8)	0.5 (0.4-0.6)
Weekly team sports (not ice hockey)			
No	1	1	1
Yes	1.5 (1.3-1.8)	1.0 (0.9-1.1)	1.8 (1.6-2.0)
Weekly weight training			
No	1	1	1
Yes	1.2 (1.0-1.4)	0.9 (0.8-1.0)	1.2 (1.1-1.4)
Weekly combat training			
No	1	1	1
Yes	0.7 (0.5-1.0)	0.9 (0.8-1.1)	1.0 (0.8-1.2)
Weekly skiing			
No	1	1	1
Yes	0.6 (0.5-0.8)	0.8 (0.7-0.9)	0.5 (0.3-0.6)
Weekly ice hockey			
No	1	1	1
Yes	1.6 (1.4-1.9)	1.0 (0.9-1.1)	2.0 (1.8-2.3)
Weekly downhill skiing			
No	1	1	1
Yes	1.0 (0.8-1.2)	1.4 (1.2-1.6)	1.3 (1.2-1.6)

*Adjusted for urbanization level of residence, childhood family composition, level of education, leisure-time sports activity, self-reported physical fitness, and age

Finally, we examined whether the associations between tobacco products use and sports activity changed over time (1999-2010). The associations between tobacco product use (snus, tobacco, dual use) and physical activity did not significantly change over time (data not shown).

## Discussion and Conclusions

The main finding of the study was that exclusive use of snus is associated with higher intensity sport activity. Respondents with higher sports activity reported lower odds ratios for cigarette smoking than those not engaged in sports. Dual use seemed to be more common among males actively engaging in sports than among those who do not engage in sports. In particular, persons training several times a week in a team sport like ice hockey, reported higher prevalence of snus use and dual use.

Possible explanations for increased snus use among physically active persons are that snus use is perceived as a less health-damaging form of tobacco use, it is thought to be less harmful to oxygen uptake compared to smoking, or that its negative health effects are poorly known. Sports requiring more individual performance and maximal oxygen intake (e.g., running, swimming, cycling, and cross-country skiing) were not associated with any tobacco product use. Consistent with previous studies [7,15], we found that snus use was related to physical activity organized by sports clubs or teams suggesting that snus use is somehow promoted or even distributed in these environments. These issues must be more thoroughly investigated in further studies. In the sphere of team sports, peer relationships and social support are crucial components. Based on theories of health behaviours, social norms are important in determining the behavior of individuals [16,17]; if individuals perceive a certain behaviour to be common among their peers and feel that it is important to conform to social norms, they are more likely to engage in that behaviour themselves than would otherwise be the case.

When joining the EU in 1995, Finland applied for a total ban on snus sales, which explains why access to snus is more difficult than access to cigarettes. Snus is, however, easy to import for personal use from neighbouring Sweden, where manufacturing and selling of snus is legal [11]. "Personal use" is defined by Finland as such a large number of snus packages, however, that it is possible to organise illegal sales of snus [11], particularly when cruises to Sweden are common for Finns and snus is sold on the ferries.

During the study period (1999-2010), cigarette smoking decreased while snus use and dual use increased. It has been suggested that people, especially those actively participating sports, have changed from cigarette smoking to snus use. Based on our findings during the 11 years, however, the association between sports activity and smoking, snus use, or dual use remained stable. At least in young people who are just beginning their tobacco-use career, switching from one product to another does not seem to be important. The reasons for the increased snus use and decreased smoking in Finland remain largely unclear. Additional research is needed to determine the reasons for this development and whether snus is used as a method to quit smoking or whether snus use reflects changes in an individual's other lifestyle behaviours, such as physical activity.

The major limitations of the study are related to self-reporting and a cross-sectional study design. Although we cannot exclude the possibility of under- or over-reporting, studies have shown that, in general, youth provide valid reports of their tobacco use [18] whereas they tend to overestimate physical activity [19]. An important limitation of the present study is that military service is completed by approximately 80% of the age cohort and thus, despite the excellent response rate, the study population was slightly selected. It should be taken into account that 20% of this age group is suspended from military duty for medical reasons or choose civilian service. Another limitation is that we could not assess the exact frequency of snus use or number of cigarettes smoked using the questionnaires. It is, unlikely, however, that this would affect the comparison between different activity groups. Because of the cross-sectional design, causal inferences cannot be drawn from our results. We cannot conclude whether young boys learn the use of different tobacco products after engaged in e.g. team sports or whether there is a selection of different smokers or non-smokers to different sports activities. It is not possible to conclude either if different tobacco product use is adopted inside or outside the sports activities. Despite these limitations, this study has notable strengths. The study had a high response rate and is unique because we were able to test the evolution of the associations between tobacco products use and physical activity over a long period of time (1999-2010).

In conclusion, our findings indicate that the use of snus is associated with higher sports activity and that persons with higher sports activity report lower cigarette use than those not engaging in sports. In particular, persons training several times a week in ice hockey or other team sports reported high levels of snus use and dual use. Sport events requiring maximal oxygen intake (e.g., running, swimming, cycling, and skiing) were not associated with tobacco product use. Although the association between snus use and team sports requires more research, preventive measures concerning tobacco product use may be targeted to sports clubs.

## Competing interests

The authors declare that they have no competing interests.

## Authors' contributions

VM carried out the statistical analysis and wrote the first draft of the manuscript with SR. HP was responsible for the initiation of the survey and he also participated the data analysis and writing. MM participated the design and coordination of the study with AR. All authors have read and approved the final manuscript.

## Pre-publication history

The pre-publication history for this paper can be accessed here:

http://www.biomedcentral.com/1471-2458/12/230/prepub
